# The movement imperative: reimagining healthcare through the lens of human movement

**DOI:** 10.3389/fspor.2026.1667847

**Published:** 2026-05-07

**Authors:** Madhur Mangalam, Jessica Fabianiak

**Affiliations:** Department of Biomechanics, University of Nebraska at Omaha, Omaha, NE, United States

**Keywords:** aging, exercise prescription, frailty, functional assessment, movement-centered healthcare, physical activity

## Abstract

Modern healthcare systems prioritize diagnostic technologies such as blood tests, imaging, and genetic screening, while systematically neglecting the fundamental human capacity for movement. This paradox is especially striking given that movement is the primary evolutionary function of the human body and a powerful predictor and determinant of health outcomes across the lifespan. Growing evidence suggests that limitations in movement capacity initiate a cascade of physiological deterioration that manifests as frailty, cognitive impairment, and premature mortality. Yet despite its centrality to healthy human function and lifespan, movement remains peripheral in clinical practice, often evaluated informally or only after a significant decline has occurred. Here, we argue for a movement-centered healthcare infrastructure that places mobility assessment and intervention at the core of clinical decision-making. We examine the scientific rationale, economic consequences, and broad societal benefits of prioritizing locomotion alongside traditional diagnostics. Ultimately, we propose that movement analysis should be elevated to the level of routine, sophisticated practice—on par with today’s laboratory diagnostics—restructuring healthcare around the proactive enhancement and lifelong preservation of human mobility.

## 1 Introduction: the paradox of sedentary medicine

In stillness, the body forgets its purpose; in movement, it remembers its power.

Contemporary healthcare operates within a profound structural contradiction. While our diagnostic capabilities have expanded dramatically—enabling detection of subtle biochemical shifts, high-resolution anatomical changes, and single-nucleotide genomic variations—we systematically overlook assessment of the most foundational human function: movement (1, 2). This is not merely a clinical blind spot but a conceptual failure to recognize that health expresses itself not only in molecules and images, but in coordinated, adaptive action. The irony is stark: we can sequence entire genomes within hours, yet lack standardized methods to evaluate the complex, integrated behavior those genes ultimately support. Movement is not peripheral to human physiology—it is its primary evolutionary purpose and a central determinant of health across the lifespan (3, 4). Yet it remains marginal in both diagnostic protocols and care pathways.

The human body evolved over millions of years under relentless selective pressure specifically for locomotion. Every physiological system—from cardiovascular networks delivering oxygen to working muscles, to neurological pathways coordinating complex motor patterns—has been shaped by the imperative to move efficiently across varied terrain (5–8). Yet our medical infrastructure treats movement as peripheral, relegating it to rehabilitation services accessed only after pathology manifests and decline begins (9, 10). In privileging molecular and anatomical insights over functional assessment, we have built clinical systems around a core misunderstanding: that human beings are biochemical systems to be corrected rather than moving organisms to be sustained. Healthcare without movement assessment is as incomplete as cardiology without electrocardiograms.

Our argument applies most directly to non-communicable diseases constituting over 70% of global mortality—cardiovascular disease, metabolic syndrome, neurodegenerative conditions, and cancers of affluence (11). Yet implications extend further. Even in acute contexts—infectious disease, surgical recovery, trauma—baseline movement capacity directly shapes outcomes (12, 13). The cancer patient’s chemotherapy tolerance, the elderly patient’s pneumonia survival, the trauma victim’s rehabilitation trajectory: each is mediated by functional reserve built over a lifetime of movement (14, 15). We do not propose that movement replace antibiotics or chemotherapy, but that it occupy the central organizing role in a healthcare infrastructure currently built around reactive, disease-specific intervention.

## 2 The biological primacy of movement

Six million years of running. Sixty years of sitting. The body has not forgotten for which it was made.

Human evolution occurred exclusively in environments demanding extensive daily movement for survival, shaping every aspect of our biological architecture over millions of years. Our ancestors engaged in complex locomotor behaviors for 6–8 hours daily—walking, running, climbing, carrying, and manipulating objects across terrain that would challenge modern athletes (16–19). This evolutionary heritage established an obligatory relationship between physical activity and health. The human genome itself represents a blueprint optimized for movement, with thousands of genes dedicated to supporting locomotor function (20–22)—genes that remain active, awaiting the mechanical signals that walking and running once reliably provided.

Evolutionary mismatch provides a powerful framework for understanding why modern sedentary lifestyles generate widespread health consequences across populations. When organisms encounter environments dramatically different from those that shaped their evolution, dysfunction results as biological systems fail to operate within their evolved parameters (5–8). Sedentary lifestyles represent perhaps the most significant mismatch in human experience, violating the basic assumptions upon which our physiology operates. This mismatch operates at every level of biological organization, from cellular metabolism to organ system function. The result is a disproportion that puzzles intuition: how can the mere absence of movement—the most passive state imaginable—produce such aggressive pathology?

### 2.1 Mechanotransduction: the cellular requirement for mechanical input

The answer partly lies in mechanobiology, a field revealing how cells depend on mechanical stimulation to maintain normal function. Cells do not passively await chemical signals; they actively sense and respond to physical forces through mechanotransduction (23, 24). Mechanosensitive proteins—including integrins at cell-matrix junctions, Piezo ion channels in cell membranes, and YAP/TAZ transcriptional regulators shuttling between cytoplasm and nucleus—convert physical deformation into biochemical signals governing gene expression, metabolic activity, and cell fate (25, 26). This is not peripheral regulation; it is fundamental to cellular function.

When mechanical stimulation ceases, mechanotransduction pathways fall silent, and the cellular processes they regulate begin to degrade (3, 27). Mitochondrial biogenesis slows (28, 29). Inflammatory signaling shifts toward chronic activation (30, 31). Muscle protein synthesis decreases while proteolytic pathways accelerate (32, 33). Bone-forming osteoblasts reduce activity while bone-resorbing osteoclasts continue unabated (34, 35). These are not gradual accommodations but pathological cascades initiated by loss of signals cells have evolved to expect. The emerging field of mechanomedicine positions mechanical forces as key modulators of health and disease, with therapeutic implications spanning oncology, cardiovascular medicine, and neurodegeneration (36–38). At the molecular level, the body does not merely benefit from movement—it requires it.

### 2.2 Myokines: muscle as an endocrine organ

The discovery that skeletal muscle functions as an endocrine organ explains why movement exerts such broad systemic effects (39, 40). When muscles contract, they release myokines—a family now including hundreds of signaling proteins, peptides, and metabolites—that travel through the bloodstream to communicate with brain, liver, adipose tissue, pancreas, bone, and immune system (41). This muscle–organ crosstalk provides a molecular explanation for otherwise inexplicable phenomena: how contracting leg muscles simultaneously reduce depression, improve insulin sensitivity, modulate immune function, and suppress tumor growth.

Muscle is far more than mechanical tissue for generating force; it is a master regulatory organ whose secretome orchestrates systemic physiology. Interleukin-6, released during contraction, enhances glucose uptake, stimulates fat oxidation, and exerts anti-inflammatory effects distinct from pro-inflammatory IL-6 produced in chronic disease (42, 43). Brain-derived neurotrophic factor promotes neuroplasticity and neurogenesis (44, 45). Irisin induces browning of white adipose tissue, increasing energy expenditure (46, 47). Myostatin, whose expression decreases with exercise, normally inhibits muscle growth—its suppression permits hypertrophy and metabolic improvement (48, 49). These signals create a molecular language through which muscle contraction communicates the body’s activity state to every organ.

Inactivity silences this network. When muscles do not contract, myokine secretion diminishes, and distant organs are deprived of signals they evolved to expect (50, 51). The sedentary body is not merely under-exercised but under-signaled, its organs operating without the regulatory input that coordinates their function. This reframes physical inactivity not as a lifestyle choice with health consequences but as a form of endocrine deficiency affecting every organ system (3).

### 2.3 Systemic integration: why movement affects everything

Movement functions as a master regulator of human physiology because mechanotransduction and myokine signaling operate across every major biological system simultaneously. At the metabolic level, regular physical activity governs glucose homeostasis, lipid oxidation, insulin sensitivity, and mitochondrial biogenesis through pathways requiring continual activation (52, 53). The cardiovascular system depends on consistent mechanical loading for cardiac output, vascular elasticity, and endothelial health (54). The brain requires movement-induced increases in cerebral perfusion, BDNF secretion, and neurogenesis to maintain cognitive function and emotional regulation (55, 56). The musculoskeletal system remodels continuously in response to mechanical loading as described by Frost's mechanostat (34). The immune system calibrates its inflammatory tone based on signals from contracting muscle (57).

Because these systems are deeply interconnected, movement deficiency does not stay local—it spreads across physiology. Muscular weakness propagates into metabolic dysregulation; cardiovascular strain compounds neurological vulnerability; immune dysregulation accelerates tissue degeneration. The failures cascade (58). Cardiorespiratory fitness shows dose-response relationships with protection against multiple cancer types, cognitive decline, and all-cause mortality—effects remaining significant even after adjustment for adiposity and traditional risk factors (59–61). Movement is not a lifestyle choice modulating health at the margins. It is a biological requirement, and the body enforces this requirement whether we acknowledge it or not.

## 3 Movement decline as the primary aging pathway

Aging begins not when cells divide slowly, but when steps grow short.

The aging process may be initiated not by molecular wear or isolated organ dysfunction, but by an early, often subtle, decline in movement capacity. This movement-centered model reframes senescence not as disease-specific pathology but as a systemic trajectory of functional loss unfolding predictably across physiological systems (62–64). From this perspective, aging is not inevitable multiorgan degeneration but the consequence of progressive disruption in mobility—a function central to our evolutionary design. This reconceptualization challenges the current compartmentalized approach to aging, positioning movement not as a late-stage casualty but as its earliest driver and clearest signal—and therefore the primary target for intervention.

The clinical syndrome of frailty—characterized by slowness, weakness, low physical activity, and exhaustion—exemplifies this movement-centric decline. Rather than emerging abruptly, frailty evolves through a self-reinforcing cycle triggered by any event that reduces physical activity: injury, illness, pain, or gradual deconditioning. This reduction initiates sarcopenia, the rapid loss of muscle mass and strength, which can begin within days of inactivity and accelerates with ongoing disuse (65–68). As capacity declines, individuals reduce activity further, compounding weakness and diminishing resilience across cardiovascular, metabolic, and neurological systems. What appears clinically as sudden frailty is the endpoint of years of subclinical deterioration.

This cascade is increasingly recognized as *disuse syndrome*—a systemic physiological deterioration directly attributable to insufficient mechanical loading (69–71). Disuse precipitates cardiovascular deconditioning, reducing maximal oxygen uptake and cardiac output by up to 25% within weeks (72, 73). Simultaneously, muscle atrophy proceeds at 0.5%–1% per day under immobilization (33). Bone loss accelerates by an order of magnitude compared to normal aging trajectories (74, 75). The metabolic consequences are equally pronounced, with impaired glucose tolerance and lipid metabolism emerging as regulatory mechanisms degrade with inactivity.

The speed and breadth of these changes highlight the fragility of human physiological equilibrium and the central role of movement in preserving it. Bed rest studies in healthy young adults show significant physiological deterioration within days. Cardiovascular fitness, muscle mass, bone density, and insulin sensitivity all decline rapidly, often requiring extended rehabilitation to recover (76). Notably, these losses occur much faster than gains achievable through exercise, revealing a biological asymmetry that favors decline over recovery (77, 78). The asymmetry is stark: three days of bed rest can undo three weeks of training. The imbalance only worsens with age.

Movement-based metrics have emerged as exceptionally sensitive and integrative indicators of overall health status and long-term longevity. Gait speed, often called the “sixth vital sign,” shows strong and consistent correlations with survival rates, hospitalization risk, functional independence, and quality of life—often outperforming conventional clinical diagnostics (79–81). A 0.1 m/s decrement in walking speed is associated with a 12% increase in mortality risk (81). Similarly, grip strength—a low-cost, rapid test of muscular function—predicts mortality as accurately as, or better than, conventional cardiovascular markers such as blood pressure (85–87). The exceptional prognostic value of these simple measures suggests something profound: a six-minute walk test may reveal what an MRI cannot—whether the body’s systems are working together or falling apart.

## 4 The diagnostic gap: movement assessment in healthcare

We measure the chemistry of blood with precision, yet ignore the poetry of motion that sustains life.

Modern healthcare operates within a diagnostic paradigm shaped by reductionist assumptions—fragmenting the human body into discrete systems, organs, and biomarkers while routinely neglecting integrated function (88). Standard evaluations include extensive blood chemistry panels quantifying lipids, glucose, inflammatory cytokines, hormones, and vitamins with molecular precision. Imaging modalities—radiography, CT, MRI, ultrasound—offer high-resolution anatomical insights, while genetic sequencing reveals disease predispositions and guides pharmacogenomic interventions. Functional diagnostics such as electrocardiography, cardiac stress testing, and pulmonary function assessments complete a technologically sophisticated diagnostic arsenal capable of detecting subtle early deviations from physiological norms across multiple systems.

Yet within this diagnostic infrastructure, a critical omission persists: systematic evaluation of movement is absent from routine medical care across nearly all clinical domains (89). Sophisticated tools for analyzing movement—motion capture, force platforms, wearable sensors—exist in biomechanics laboratories and elite sports facilities but remain siloed from primary care and general medicine. This disconnect is especially paradoxical given that movement metrics can rival or surpass conventional biomarkers in predicting morbidity, mortality, and functional independence, while requiring no invasive procedures, expensive reagents, or ionizing radiation (79–87). The healthcare system can detect microvariations in enzyme levels but fails to assess gait, posture, or physical adaptability—functions at the heart of human health. The oversight runs deeper than workflow or equipment; it reflects what medicine implicitly believes bodies are for.

This diagnostic blind spot is rooted in foundational healthcare delivery structures that prioritize disease identification and pharmaceutical intervention over prevention and functional optimization. Medical curricula dedicate disproportionate attention to pathophysiology and pharmacology, offering little formal education in movement science, exercise physiology, or functional assessment. A systematic review from 2002 found that only 13% of U.S. medical schools included physical activity curricula, with most programs offering fewer than four hours of instruction across the entire medical education (90). More recently, a 2022 consensus statement from sports medicine physicians outlined comprehensive curricular recommendations—including exercise prescription, motivational interviewing, and community resource navigation—yet implementation remains inconsistent (91). Clinicians graduate with robust training in disease mechanisms but limited capacity to evaluate or prescribe movement-based interventions. This epistemic gap fosters a healthcare culture in which movement is addressed reactively—after injury or illness, not proactively as a central health determinant. The net effect is a workforce prepared to prescribe a panoply of drugs but untrained in leveraging one of the most powerful therapeutic tools available: movement itself.

Movement assessment is not entirely absent from healthcare. The International Classification of Functioning, Disability, and Health (ICF), endorsed by all 191 WHO Member States in 2001, provides conceptual architecture for understanding functioning through a biopsychosocial lens (92). World Physiotherapy adopted the ICF framework in 2003, and rehabilitation medicine has incorporated functional assessment into specialty practice. Yet these frameworks remain confined to post-hoc settings, accessed only after disease or injury has manifested. They have not been integrated into primary care as a routine evaluation. A 2023 survey of athletic trainers found that 51.9% had never learned about the ICF framework, with low implementation across all assessment categories (93). A 2024 study concluded that ICF education alone does not translate into clinical implementation without specific practical training (94). The ICF offers architecture; what we lack is construction.

When movement is assessed clinically, it is typically reduced to imprecise proxies such as self-reported activity or isolated functional tests administered only in specialized settings (95–97). In contrast to precision in other health domains, movement is crudely measured, if at all. Meanwhile, advanced methods—three-dimensional kinematics, kinetic force analyses, surface electromyography—remain underutilized in clinical practice despite their availability and technical maturity (98–100). Validated wearable devices offer additional promise by enabling continuous, real-world movement monitoring (101, 102), yet they too are rarely integrated into routine clinical workflows. These technologies could revolutionize health assessment, transforming how we detect risk, monitor recovery, and tailor interventions. That such technologies exist yet remain largely excluded from medical decision-making constitutes a striking missed opportunity. Clinicians assess lipid panels to two decimal places while remaining unable to describe how their patients walk (103–105).

This diagnostic vacuum is no longer a technological necessity—it is an infrastructural choice. The past decade has witnessed a revolution in wearable sensor technology, transforming devices once limited to step counts and heart rate into clinical-grade instruments capable of capturing subtle movement dysfunction signatures. The Mobilise-D consortium has validated 24 digital mobility outcomes across six clinical cohorts spanning Parkinson’s disease, multiple sclerosis, chronic obstructive pulmonary disease, hip fracture recovery, heart failure, and healthy aging—demonstrating that real-world gait speed, stride variability, and walking bout characteristics can be measured with accuracy approaching laboratory gold standards (106, 107). Consumer-grade devices now achieve sufficient precision for meaningful clinical applications, with systematic reviews confirming acceptable validity for gait parameters in both controlled and free-living conditions (108). Technology has solved the measurement problem. What remains is the will to use it.

## 5 Economic and social dimensions of movement suppression

The architecture of our cities shapes the biology of our bodies, one sedentary choice at a time.

The suppression of movement in modern society goes beyond individual choices or clinical neglect. It is a systemic condition, deeply embedded in the design of our cities, the structure of our economies, the rhythms of our work, and the values of our culture. It reflects a multilayered ecosystem of constraints producing what researchers term “obesogenic” or “pathogenic” environments: social and physical contexts that actively promote sedentary behavior, physiological dysfunction, and widespread disease (109–112). Technologies intended to enhance human well-being have paradoxically constructed ecosystems that erode the behaviors most essential to health.

Urban design provides one of the manifestations of this paradox. Over the past century, planning decisions have privileged motorized transportation at the expense of human-powered mobility, producing sprawling suburbs and car-dependent cities that render walking and cycling impractical, unsafe, or socially marginalized (113, 114). The built environment becomes a silent architect of chronic disease. In such contexts, movement becomes an optional, time-consuming activity segregated into narrowly defined “exercise sessions” (115–117)—a pattern that violates the integrated functional movement for which our physiology evolved. When environments are built to restrict movement, even the most motivated individuals struggle to sustain health-supporting behaviors.

Economic structures reinforce these constraints through sweeping shifts in occupational patterns. The transition from labor-intensive manufacturing and agriculture to knowledge- and service-based economies has transformed most workplaces into sedentary spaces where prolonged sitting is the norm and physical activity is both unnecessary and logistically difficult (118–120). For many adults, the workplace is where most waking hours are spent, and those hours are increasingly defined by inactivity—often in ergonomically suboptimal environments designed for productivity rather than physiological well-being (121, 122). The modern economy systematically violates human evolutionary imperatives in exchange for productivity, generating health problems that the same system must then pay to treat.

The economic consequences of movement suppression are staggering. The WHO’s 2022 Global Status Report on Physical Activity estimated that physical inactivity will cost global healthcare systems $300 billion by 2030 if current prevalence remains unchanged (124), with 74% of new diabetes cases and 76% of new ischemic heart disease cases attributable to insufficient activity occurring in low- and middle-income countries—yet 63% of direct healthcare costs will be borne by high-income nations (123). Yet direct medical costs represent only a fraction of the broader economic burden. Indirect costs—lost productivity, absenteeism, presenteeism, disability, and premature mortality—exert even greater strain, siphoning human potential and reducing collective well-being at scale (125). The burden falls disproportionately on economically and socially marginalized populations, exacerbating existing inequities in health, income, and opportunity. Movement suppression functions as both a public health crisis and a structural determinant of inequality.

Cultural norms and social expectations compound these structural challenges by reinforcing the desirability of convenience, efficiency, and technological substitution for physical effort. Sedentary behaviors are valorized as signals of productivity or prestige, while physical exertion is often stigmatized as burdensome, inefficient, or unnecessary (126–128). The medicalization of aging-related mobility decline frequently results in premature activity restriction over proactive interventions to maintain or restore function, thereby hastening physical deterioration through well-intentioned but misguided caution (129–131). Meanwhile, the pervasive influence of digital entertainment and algorithmically optimized platforms has created unprecedented competition for attention, making the delayed benefits of physical activity pale in comparison to instant gratification offered by screens. The result is a culture that systematically devalues movement while promoting the behaviors most predictive of chronic disease and functional decline across the lifespan (132–134).

Movement suppression is not simply a medical oversight—it is a systemic pathology embedded in the scaffolding of modern life, and addressing it will require coordinated reform across infrastructure, economics, and culture.

## 6 Evidence base for movement-centered interventions

In the laboratory of human experience, movement is both the hypothesis and the proof.

The scientific foundation for such reform has grown across virtually every domain of medicine, demonstrating not only wide-ranging clinical efficacy but also the ability to address multiple physiological systems simultaneously—often with fewer risks and greater reach than conventional pharmacological interventions. Movement interventions have shown effectiveness in preventing and treating chronic diseases, improving mental health and cognitive function, reducing cancer risk, enhancing musculoskeletal integrity, and optimizing sleep—all while incurring minimal side effects. Unlike most medical therapies, which act on narrow physiological targets, physical activity exerts global regulatory effects on human biology, reflecting its evolutionary centrality. Despite the consistency and breadth of the evidence, movement remains underutilized in clinical settings—revealing a gap not in knowledge but in implementation. Bridging this divide is among the most urgent challenges facing modern healthcare systems.

Cardiovascular disease prevention is perhaps the clearest domain where movement interventions rival or exceed pharmacologic strategies. Regular physical activity reduces cardiovascular risk by 30%–50% across populations, with benefits evident even at modest activity levels and increasing with greater intensity and duration in a clear dose-response pattern (135–138). These effects operate through multiple pathways—lipid metabolism, blood pressure regulation, endothelial function, insulin sensitivity, and inflammatory modulation. Physical inactivity is now recognized as a modifiable risk factor on par with smoking, hypertension, and dyslipidemia (139, 140). The consistent efficacy of movement across age, sex, and comorbidity profiles underscores its suitability as a first-line cardiovascular intervention. The prescription pad has room for one more line.

The case for movement in diabetes prevention and management is similarly compelling. The Diabetes Prevention Program demonstrated that lifestyle interventions emphasizing physical activity reduced diabetes incidence by 58%, outperforming metformin, which yielded only a 31% reduction (141). Beyond glucose regulation, exercise improves cardiovascular health, quality of life, and physical capacity, while enabling reductions in medication use and their associated costs and side effects (142, 143). These results have elevated structured physical activity to a core component of professional guidelines for type 2 diabetes. The breadth of additional benefits and absence of adverse effects make movement a uniquely potent and underutilized factor in metabolic disease management.

Mental health is another domain where movement interventions are redefining therapeutic norms. Exercise shows antidepressant and anxiolytic effects on par with—or in some cases superior to—pharmacological treatments, especially when adherence and side effect profiles are considered (144). Meta-analyses consistently report reductions in symptoms of depression and anxiety across demographic groups and clinical contexts (145–147). Mechanistically, movement enhances neuroplasticity, modulates neurotransmitter systems, and fosters social connection and stress resilience—all critical for sustained psychological health. Given the limitations and escalating use of psychiatric medications, movement-based approaches offer something increasingly rare: a low-cost, high-access intervention that addresses root causes rather than masking symptoms.

Physical activity shapes cognitive function and brain health across the lifespan. Exercise enhances executive function, memory, and processing speed, with effect sizes comparable to cognitive training interventions (148, 149). Underlying mechanisms include increased brain-derived neurotrophic factor, enhanced cerebral blood flow, reduced neuroinflammation, and stimulation of hippocampal neurogenesis (150, 151). These effects position physical activity as the most promising non-pharmacological strategy for preventing or delaying cognitive decline and dementia. This is especially urgent given the global rise in age-related neurodegenerative diseases and the limited efficacy of current pharmaceutical treatments.

The evidence base for cancer prevention and survivorship has been substantially strengthened by recent meta-analytic work. Regular physical activity reduces the risk of several major cancers, including breast, colorectal, lung, and prostate, with relative risk reductions of 10%–25% depending on type and dose (152–154). A 2025 systematic review in the *British Journal of Sports Medicine* analyzing 42 studies with over 46,000 cancer patients demonstrated that high muscle strength and cardiorespiratory fitness were associated with 31%–46% reduced risk of all-cause mortality across cancer types—including in patients with advanced disease stages (155). Critically, each one-MET increment in cardiorespiratory fitness was associated with an 18% reduction in cancer-specific mortality. These magnitudes rival or exceed those achieved by many oncological therapies, yet movement interventions remain peripheral in cancer care pathways. For individuals undergoing treatment, movement improves chemotherapy tolerance, mitigates fatigue, and enhances physical and emotional quality of life (156, 157). Post-treatment, it may also improve survival outcomes through mechanisms such as immune modulation, reduced systemic inflammation, hormonal regulation, and enhanced physical resilience. These benefits make the case plainly: in oncology, movement is not supportive care. It is care.

Musculoskeletal health offers perhaps the clearest illustration of movement’s preventive and restorative power. Resistance training and weight-bearing exercises increase bone density, muscle mass, and neuromuscular coordination even in very elderly populations (158–160). These adaptations reduce fracture risk and enhance mobility, independence, and quality of life. Falls prevention programs based on strength and balance training consistently reduce fall incidence by 20%–30% in high-risk populations (161–163). As pharmacologic approaches to sarcopenia and osteoporosis remain limited and often poorly tolerated, movement remains the most accessible and effective strategy for preserving structural resilience and preventing disability in aging populations.

Sleep, often underappreciated as a clinical endpoint, improves with regular movement. Physical activity reduces sleep onset latency, improves sleep efficiency, and enhances sleep quality while reducing dependence on sedatives and other sleep aids that carry side effects and addiction risks (164–166). Improved sleep, in turn, amplifies daytime energy, cognitive function, immune regulation, and emotional well-being (167–169). Better nights beget better days; the cycle is virtuous.

The COVID-19 pandemic provided a striking real-world demonstration of movement’s protective role beyond chronic disease. Systematic reviews demonstrated that physical inactivity was strongly associated with worse COVID-19 outcomes—hospitalization, ICU admission, and death (2). More strikingly, Long COVID—the post-acute syndrome affecting millions worldwide—manifests primarily as exercise intolerance, fatigue, and functional limitation. A 2024 meta-analysis found that exercise-based rehabilitation improved symptom severity, physical fitness, and quality of life in patients with Long COVID (170). The syndrome reveals what evolutionary biology predicts: an infectious insult becomes a chronic disability precisely when movement capacity is compromised, and recovery proceeds through the restoration of functional movement.

A necessary caveat accompanies this evidence. Like any intervention, movement carries risks that must be managed through individualized assessment, prescription, and monitoring. A 2024 review on exercise intensity prescription in cardiac rehabilitation emphasized that optimizing intensity is crucial to maximize clinical benefits while minimizing complications—too little yields subtherapeutic effects, while too much may precipitate adverse events in vulnerable populations (171). An umbrella review examining exercise prescription variables in musculoskeletal pain identified significant evidence gaps regarding optimal dosing parameters, underscoring that movement interventions require the same methodological rigor as pharmacotherapy (172). This is precisely our argument: not that movement is a panacea without risk, but that its professionalization within healthcare would ensure the same systematic attention to dosing, contraindications, and monitoring currently afforded to pharmaceutical agents. The risks of movement are real but bounded; the risks of inactivity are systemic and cumulative.

These domains highlight the unmatched therapeutic versatility of movement across the continuum of health and disease. Few, if any, clinical interventions offer such widespread, evidence-based benefit with such minimal risk. The evidence is settled. What remains is recognition, funding, prescription, and the clinical seriousness we already extend to pharmaceuticals.

## 7 Toward movement-centered healthcare infrastructure

The future of medicine lies not in treating disease, but in cultivating the movement that prevents it.

Reorienting healthcare around this evidence demands fundamental rethinking of clinical practice, professional training, health financing, and care delivery models. This transformation extends beyond technical development of movement diagnostics—it necessitates systemic changes that address the structural, cultural, and institutional barriers that have long marginalized movement science within mainstream healthcare. The scope of this shift is not incremental but paradigmatic, requiring coordinated action across multiple levels of the healthcare ecosystem—from individual provider encounters to institutional workflows and national policy frameworks (173–175). Overcoming decades of professional silos and institutional inertia is essential—but so is something simpler: learning to see movement as central to medicine rather than incidental to it.

At the core of this infrastructure is developing standardized, clinically valid movement assessment protocols that achieve the reliability and diagnostic power of contemporary laboratory tests (176–178). These protocols must capture multidimensional aspects of movement—quality, efficiency, capacity—through validated instruments that yield interpretable and actionable clinical data. Emerging wearable sensors, smartphone-based monitoring, and AI-driven analytics now offer unprecedented opportunities to extend precision assessment capabilities from biomechanics laboratories into routine clinical settings (101, 102).

A comprehensive movement assessment should encompass multiple domains of locomotor function. Basic measures such as gait speed, balance, and coordination offer prognostic insights across populations and conditions (79, 81–84). Muscle strength and power assessments provide critical data on functional reserve and injury risk, while cardiorespiratory fitness remains one of the most direct and integrative evaluations of physiological health under stress (179, 180). Flexibility and joint range of motion assessments identify biomechanical constraints, asymmetries, and neuromuscular inefficiencies—often before pain or disability emerges (181, 182). Together, these components form the foundation of a comprehensive functional profile that rivals conventional diagnostic paradigms in breadth and clinical utility.

Educational transformation is equally essential. Medical education at all levels must be restructured to position movement science and functional assessment as core competencies rather than elective topics (183, 184). This shift requires redefining what it means to be a competent healthcare provider in the 21st century: not only a diagnostician of disease but a steward of function.

Financial infrastructure must also evolve to support movement-based care. Current reimbursement models overwhelmingly favor acute treatment and procedural interventions while disincentivizing preventive, behaviorally rooted strategies (185, 186). Value-based payment models should reward providers for preserving function, preventing decline, and improving quality of life—not just for managing disease after it has compromised health. Reform at the institutional, state, and federal levels is necessary to align economic incentives with the biological realities of human health.

Service delivery models should reflect this emphasis on movement by embedding movement professionals—physical therapists, exercise physiologists, kinesiologists—within interdisciplinary care teams (187–189). Movement expertise should be a frontline resource embedded in preventive care, not a specialty accessed only after decline. This integration will require new professional roles, collaborative scopes of practice, and shared decision-making frameworks that break down longstanding disciplinary boundaries while preserving accountability (190, 191).

Finally, digital infrastructure must treat movement data as first-class clinical information. Electronic health records should capture, analyze, and visualize movement metrics with the same granularity currently afforded to laboratory values and imaging reports (192–194). Clinical decision support, telehealth-based movement coaching, and population-level functional surveillance all depend on this foundation (195–197). The key design principle is interoperability; movement data must be seamlessly embedded in clinical workflows—as foundational infrastructure, not afterthoughts grafted onto existing systems.

## 8 Implementation strategies and future directions

The science of movement is still writing its first chapter—but already it rewrites the story of medicine.

Implementing movement-centered healthcare is a multidimensional challenge demanding coordination across all layers of the healthcare system. From the micro-level of patient–clinician encounters to the macro-level of national policy reform, this transformation requires aligning technical, educational, economic, and cultural systems in service of a radically different paradigm of care (198–200). Success will depend not only on overcoming institutional inertia but also on articulating a clear value proposition—one demonstrating measurable improvements in health outcomes, provider effectiveness, and system-level cost savings. Early implementation efforts must be strategic, targeted, and sequenced to generate momentum through achievable wins while laying groundwork for broader systemic change. Without such deliberate efforts, healthcare will remain locked in a reactive model that treats the consequences of movement loss rather than preventing its onset.

A pragmatic starting point is developing and deploying pilot programs within health systems that have the infrastructure and leadership readiness to serve as demonstration sites. These pilots should focus on integrating standardized, high-quality movement assessment protocols—efficient, scalable, and comparable in reliability to existing clinical diagnostics (201–205). Protocols should be designed for use by a range of healthcare professionals, require minimal equipment, and be sensitive enough to detect early signs of functional decline. Ideal pilot settings include institutions with interdisciplinary teams, digital health infrastructure, and a culture open to innovation. Evidence generated from these pilots will be instrumental in validating clinical utility and economic feasibility, providing the proof points needed for expansion to more complex or resource-constrained environments.

Early-stage implementation should also prioritize populations where movement interventions deliver the most pronounced impact. Older adults, who face high risks of hospitalization, institutionalization, and functional decline, constitute an ideal focus given substantial evidence supporting movement interventions to improve independence and quality of life (206, 207). Similarly, individuals with chronic conditions—type 2 diabetes, cardiovascular disease, depression—often experience outcomes from movement-based interventions that rival or exceed those of pharmacologic treatment (208, 209). These high-need populations offer both compelling clinical returns and strong economic justification, especially in value-based care models (210, 211). Demonstrating success in these groups strengthens the case for policy support, payer investment, and broader population-level implementation.

Provider education is a critical enabler of long-term sustainability. Current professional training remains heavily skewed toward disease recognition and pharmacologic management, with minimal emphasis on functional assessment or physical activity counseling (212, 213). Medical, nursing, and allied health curricula must be restructured to integrate movement science not as an elective but as a core competency. This includes practical instruction in gait assessment, strength testing, and exercise prescription, supported by evidence-based frameworks and rigorous competency evaluation (214, 215). Continuing education must also address the substantial knowledge gap among practicing clinicians never trained in movement-focused approaches. Interprofessional training environments that bring together physicians, nurses, therapists, and exercise professionals are particularly valuable for fostering collaboration and dismantling the professional silos that impede integrated care (216, 217).

Technology development constitutes a particularly transformative priority. The same devices that enabled our sedentary confinement now offer instruments of liberation. Wearable sensors and mobile health platforms have matured from consumer novelties to clinical-grade technologies capable of gathering continuous, real-time movement data outside laboratory settings, enabling early detection of decline and ongoing monitoring of intervention effectiveness (101, 102). As the Mobilise-D consortium’s validation across diverse clinical populations confirms, the technical infrastructure for movement-centered healthcare already exists (106). What remains is the will to deploy it.

More striking is the emerging convergence of biophysical and biochemical monitoring within single wearable platforms. Next-generation devices now enable continuous, minimally invasive analysis of sweat, interstitial fluid, breath, tears, and saliva—capturing inflammatory markers, metabolic substrates, pharmacokinetic profiles, and pathogen signatures alongside movement metrics (218). This convergence mirrors the body’s own systemic coherence: not separate streams of motion and molecular data, but a unified portrait of human function in real time. The diabetic patient’s gait variability can be correlated with continuous glucose fluctuations; the cancer survivor’s fatigue mapped against rhythms of inflammatory cytokines; the aging adult’s fall risk contextualized by hydration status and medication absorption. The devices themselves increasingly embody the holistic vision this manuscript advocates.

Artificial intelligence can process these high-volume, high-dimensional data streams to identify risk trajectories, predict outcomes, and optimize individualized prescriptions with sophistication no human clinician could achieve unaided (219–221). Digital biomarkers derived from passive smartphone and wearable data have demonstrated capacity to predict cognitive decline, detect early depression, and stratify cardiovascular risk—often with sensitivity exceeding traditional clinical assessments (222, 223). These technologies should be positioned not as replacements for human care but as decision support systems extending expert-level functional assessment into primary care and community health settings—democratizing access to sophisticated movement evaluation currently confined to research laboratories and elite rehabilitation facilities (224–226).

Parallel to technical innovation, policy research must address systemic constraints that currently marginalize movement in clinical care. Most healthcare financing models fail to reimburse evidence-based movement interventions, creating powerful disincentives to adopt them—even when they outperform pharmacological or surgical alternatives. Research should explore how revised reimbursement frameworks, value-based care models, and bundled payments could support a shift toward prevention and function-centered care. Movement assessment, exercise counseling, and physical activity prescription must be recognized and reimbursed as core clinical services rather than optional wellness add-ons.

The role of the built environment—sidewalks, parks, public transit access—in shaping population movement patterns must be rigorously studied. Behavior does not occur in a vacuum; movement is enabled or constrained by social and physical environments, and policies must reflect that reality. Governance frameworks for technology-induced sedentary behavior—including the respective roles of healthcare systems, employers, and individuals in promoting movement—represent another priority as evidence regarding screen time, remote work, and algorithmic engagement accumulates. Regulatory frameworks will be essential in defining credentials, scope, and accountability of movement professionals as they integrate into clinical teams. Addressing movement inequality—the unequal distribution of opportunities, access, and support for physical activity—should be treated as a priority on par with other public health disparities.

Personalization of movement prescriptions represents a critical frontier. Future research should investigate how individual factors—genomics, baseline motor patterns, comorbidities—can tailor exercise interventions with the precision seen in pharmacology or oncology. Experimental evaluation of integrated care models that embed movement professionals alongside traditional providers is urgently needed to determine how interdisciplinary teams can maximize functional outcomes. Cost-effectiveness studies should accompany these efforts to generate the economic evidence required for widespread adoption. Successful integration will require workflow redesign, shared communication platforms, and outcome-tracking systems that enable movement professionals and medical providers to coordinate care seamlessly. The challenge will be ensuring clinical-grade accuracy, interoperability with electronic health records, and equitable access across socio-demographic groups, languages, and levels of digital literacy.

Together, these priorities point toward a broader reimagining of healthcare itself. The evidence supports a vision in which movement is not just a therapeutic target but a vital sign, a functional biomarker, and a systems-level intervention. The human body evolved to move; its optimal function depends on movement; and when that movement is supported, health across domains—metabolic, musculoskeletal, neurological—improves at a scale rarely matched by conventional interventions. Systems that fail to assess or prescribe movement operate at odds with biological reality. Movement-centered care offers not only improved clinical outcomes but enhanced quality of life, reduced healthcare costs, and deeper alignment between medical practice and human design.

The human body is not a vessel that houses movement; it is movement crystallized in form. Every structure—bone curvature, muscle architecture, neural pathway—exists because our ancestors moved relentlessly across millions of years of evolutionary time. The genome has not changed; the mismatch is entirely of our construction. To build healthcare systems that ignore this fundamental truth is not merely misguided—it is tragic.

Yet the body remembers what the institution has forgotten. In every step, every reach, every act of rising and moving through space, the human organism expresses an evolutionary inheritance that no policy can repeal and no technology can replace. The task before us is not to invent something new but to recover something ancient—to realign our systems of care with the biological imperatives that shaped us. A healthcare system that embraces movement does not simply treat disease; it honors what the human body was built to do.

The movement imperative is, ultimately, an invitation: to build medicine worthy of the bodies it serves.
